# Rift Valley Fever Virus Infection of Human Cells and Insect Hosts Is Promoted by Protein Kinase C Epsilon

**DOI:** 10.1371/journal.pone.0015483

**Published:** 2010-11-24

**Authors:** Claire Marie Filone, Sheri L. Hanna, M. Cecilia Caino, Shelly Bambina, Robert W. Doms, Sara Cherry

**Affiliations:** 1 Department of Microbiology, University of Pennsylvania School of Medicine, Philadelphia, Pennsylvania, United States of America; 2 Penn Genome Frontiers Institute, University of Pennsylvania School of Medicine, Philadelphia, Pennsylvania, United States of America; 3 Department of Pharmacology, University of Pennsylvania School of Medicine, Philadelphia, Pennsylvania, United States of America; University of Minnesota, United States of America

## Abstract

As an arthropod-borne human pathogen, Rift Valley fever virus (RVFV) cycles between an insect vector and mammalian hosts. Little is known about the cellular requirements for infection in either host. Here we developed a tissue culture model for RVFV infection of human and insect cells that is amenable to high-throughput screening. Using this approach we screened a library of 1280 small molecules with pharmacologically defined activities and identified 59 drugs that inhibited RVFV infection with 15 inhibiting RVFV replication in both human and insect cells. Amongst the 15 inhibitors that blocked infection in both hosts was a subset that inhibits protein kinase C. Further studies found that infection is dependent upon the novel protein kinase C isozyme epsilon (PKCε) in both human and insect cells as well as in adult flies. Altogether, these data show that inhibition of cellular factors required for early steps in the infection cycle including PKCε can block RVFV infection, and may represent a starting point for the development of anti-RVFV therapeutics.

## Introduction

Viral pathogens are a common cause of morbidity and mortality in the developing and developed world. Of particular concern are emerging and re-emerging epidemic arboviral diseases that are spread by mosquitoes and other biting insects [1]. Most arboviruses impacting public health fall into one of three viral families: Flaviviridae, Togaviridae, and Bunyaviridae. Bunyaviruses are enveloped, negative-sense tripartite RNA viruses that include Sin Nombre, Hantavirus, Crimean-Congo hemorrhagic fever virus and Rift Valley fever virus (RVFV). RVFV is an important emerging pathogen due to its frequent outbreaks [2]. While humans infected with RVFV typically have a self-limited febrile illness, 1-3% die as a result of hemorrhagic symptoms [3]. RVFV was initially only endemic in sub-Saharan Africa, although the regions affected by the virus have expanded and now include Egypt and the Arabian Peninsula. In addition to *Aedes sp.*, a broad range of mosquito species has been linked to the spread of RVFV, increasing the chances that RVFV may spread to other countries [4], [5].

RVFV, and arboviruses in general, are remarkable because they have not only developed highly effective strategies to hijack cellular machinery and to subvert hosts' immune responses, but they have evolved these capabilities in broad host ranges, spanning arthropods and mammals. While infection of the insect host has little pathogenicity, infection of mammals is associated with disease. Little is known about the host factors required for the replication cycles of these viruses in either of their host genera, which impedes development of antiviral treatments.

Classically, antivirals have been developed as a result of high-throughput screens (HTS) that target specific viral enzymes. This target-based approach fails to investigate other possible targets including essential cellular factors that enable infection, or viral proteins with unknown function. Targeting cellular factors may be advantageous because such treatments are less liable to be evaded by the high mutation rate of viral genomes. In addition, the use of *Drosophila* as a model insect makes it possible to take advantage of the powerful genetic tools available in this organism to both screen and to test the role of identified targets at the organismal level [6], [7], [8], [9], [10], [11], [12], [13], [14].

We developed a cell- and image-based high-throughput screening platform that allowed us to screen a library of 1280 known biologically active small molecules for inhibitors of RVFV (strain MP12) infection in both human and *Drosophila* cells [15]. Using this strategy we identified a number of inhibitors that suppressed infection in cell lines derived from both hosts. Amongst the over-represented classes of inhibitors were drugs that are known to target macropinocytosis, including phosphatidylinositol 3-kinase (PI3K) and protein kinase C (PKC) inhibitors. Macropinocytosis is a receptor-independent endocytic mechanism that is a known entry route for some viruses, although this mechanism has not been shown to control the entry of RVFV, other bunyaviruses, or other small enveloped viruses [16], [17], [18], [19]. Further studies focused on that the role of PKC in infection. We found that the classical PKC isozymes were dispensable for infection while the novel PKC isozyme, PKC epsilon (PKCε), promotes RVFV MP12 infection. Inhibition of PKCε in human cells, *Drosophila* cells or adult flies significantly attenuated infection Together, these data show that RVFV MP12 infection of both the insect and mammalian host has conserved cellular requirements that are amenable to therapeutic intervention.

## Results

### RVFV MP12 infection of *Drosophila* and mammalian cells

**Figure 1:**
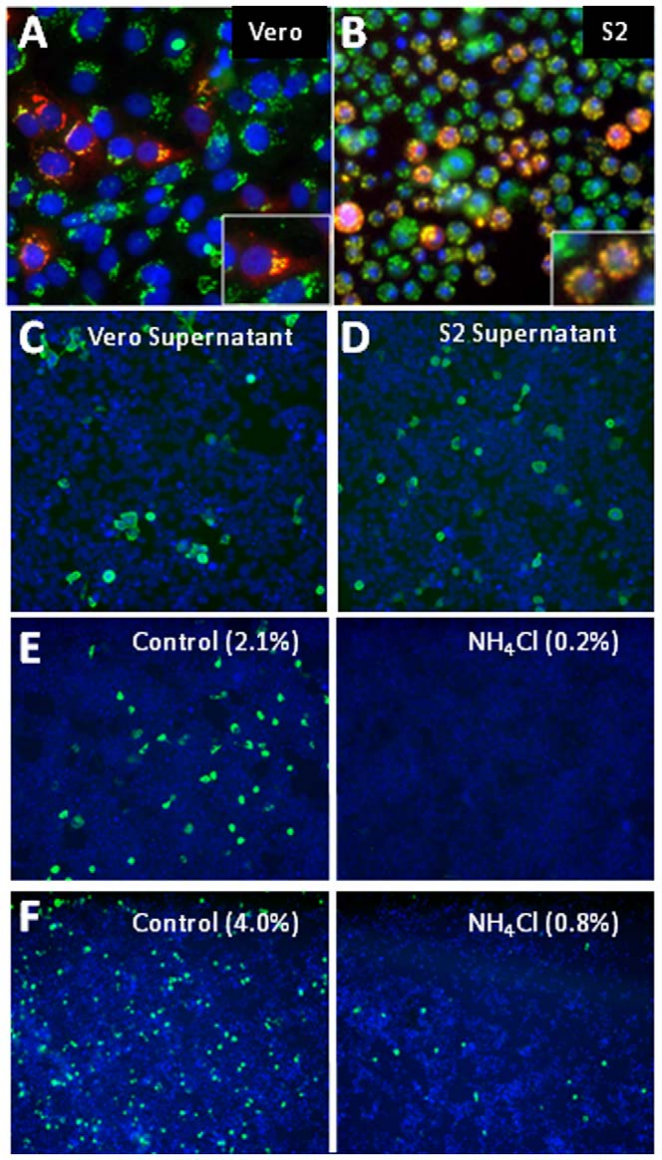
RVFV MP12 productively infects both human and *Drosophila* cells. **A**. Vero cells were infected at an MOI = 0.08 for 15 hours and RVFV infection was detected by immunofluorescence using mouse anti-RVFV G_c_ (red), anti-TGN46 (Golgi marker, green), and the nuclear dye DAPI (blue). Inset shown at higher magnification. **B**. *Drosophila* S2 cells were infected at an MOI = 0.02 for 48 hours and RVFV infection was detected by indirect immunofluorescence using mouse anti-RVFV G_N_ (red), anti-GM130 (Golgi marker, green), and the nuclear dye DAPI (blue). Inset shown at higher magnification. **C–D**. RVFV MP12 produced in Vero cells (**C**) or *Drosophila* S2 cells (**D**) was used to infect 293T/17 cells and detected by immunofluorescence with mouse monoclonal anti-RVFV N (green) and the nuclear dye DAPI (blue). **E–F**. Ammonium Chloride (NH_4_Cl; 960 µM) inhibits RVFV infection in mammalian 293T cells infected at an MOI of 0.1 for 15 hours (**E**) and *Drosophila* S2 cells (**F**) infected at an MOI of 0.1 for 48 hours. Infected cells were visualized by immunofluorescence against RVFV N (green) and counterstained with DAPI (blue).

To identify cellular factors that impact viral replication in mammalian and insect cells we used an attenuated strain of RVFV, MP12 [20]. This strain differs by 11 amino acids from the wild type strain ZH548, making it likely that cellular factors required for MP12 replication will also be needed for wild type strains [21]. We generated high-titer virus in Vero cells (10^7^ pfu/mL) and used this to infect mammalian cells including Vero, HeLa and 293T, and insect cells including mosquito C6/36 and *Drosophila* S2 cells. We found that the RVFV strain MP12 infected all cell lines tested as measured by an immunofluorescence assay in which newly produced viral G_C_ glycoproteins were detected after infection (data not shown). In Vero and *Drosophila* S2 cells, the viral glycoproteins co-localized with a Golgi apparatus marker as previously described (Figure 1A, B) [22], [23]. Importantly, RVFV MP12 infection of human and *Drosophila* cells was productive, leading to the generation and release into the media of infectious progeny (Figure 1C, D) and spread of virus in both human and insect cell cultures (data not shown), demonstrating that we can use both mammalian and *Drosophila* cells to study the entire replication cycle of RVFV.

To quantify infection, we stained cells with an antibody to a viral antigen (G_C_) and counterstained with DAPI to observe cell nuclei. Automated imaging was used to capture three sites per well in a 96 well plate, and images were analyzed using MetaXpress software to measure percent infection (G_c_^+^/DAPI^+^). We used a similar assay to monitor infection of insect cells, though infection times were longer due to slower virus replication, possibly caused by the lower temperature. Using this assay, we found that in mammalian 293T cells the lysosomotropic agent Ammonium Chloride inhibited infection by 12-fold (Figure 1E). Likewise, in *Drosophila* cells, RVFV MP12 replication was also dependent on intracellular acidification at a similar concentration (Figure 1F). This confirms that viral infection is dependent upon an acidified cellular compartment in insect and mammalian cells and shows that we can use small molecule inhibitors to probe the biology of infection in a quantitative manner.

### Screen for inhibitors of RVFV infection

To screen drugs for the ability to inhibit RVFV MP12, we developed an assay for RVFV infection of both human and *Drosophila* cells in a 384-well format. We optimized cell number for 384-well plates, viral innoculum, and staining parameters. We tested for tolerance to DMSO (<1% tolerated in both cell lines) and temperature (293T cells are robust for up to 4 hours at room temperature). To assess the extent of infection, we captured 3 sites per well for 3 wavelengths (nuclei and the viral structural proteins N and G_N_). These images were analyzed using MetaXpress software to capture the number of cells and the number of infected cells (number N^+^ and G_N_^+^). Robust statistics were used to generate the metrics used to identify outliers or potential candidate modulators. We calculated the percent infection for each well, log transformed the data, calculated the median and interquartile range from which we calculated a robust Z score plate-by-plate [24].

**Figure 2:**
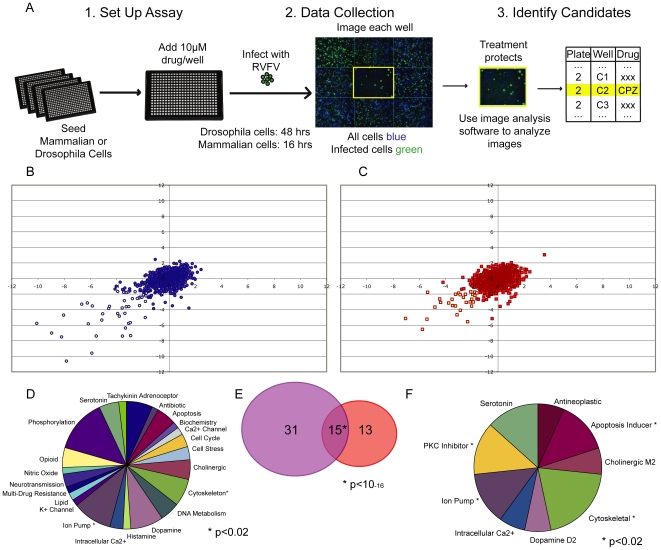
High-throughput screen for inhibitors of RVFV infection. **A**. Schematic of screening strategy used in human and insect cells. Cells are seeded in 384 well plates, subsequently 10µM drug was added. The cells were infected with RVFV, then fixed, stained and imaged using automated microscopy. Automated image analysis and statistical thresholds were used to identify inhibitors of RVFV infection. Candidate drugs are then validated and further characterized. **B**. 6,000 293T cells were plated in 384 well plates, treated with inhibitor and infected at MOI = 0.06 for 16 hours. Robust Z scores for infection in mammalian were plotted for each replicate in blue. Each of the four plates is denoted by a different symbol. **C**. 20,000 S2 cells were plated in 384 well plates, treated with inhibitor and infected at MOI = 0.02 for 48 hours. Robust Z scores for infection in mammalian were plotted for each replicate in red. Each of the four plates is denoted by a different symbol. **D**. Annotated categories of 59 candidate RVFV inhibitors from LOPAC screen. * denotes over-represented groups p<0.02. **E**. Venn diagram of the distribution of small molecules: 31 mammalian (blue), 13 insect (red) and 15 pan-inhibitors (purple). * p<10^−16^. **F**. Categories of 15 candidates that block infection of both mammalian and *Drosophila* cells. * denotes over-represented groups p<0.02.

Next, we used this assay platform to screen for small molecule inhibitors of RVFV MP12 infection both in human (293T) and *Drosophila* (S2) cells (Schematic Figure 2A). We screened a commercially available library of 1280 compounds (Sigma LOPAC^1280^) that contains marketed drugs and relevant structures with predictable activities and proven scaffolds directed against a wide range of known drug targets including GPCRs and protein kinases. We screened in duplicate for each cell type at 10 µM in 0.25% DMSO. The robust Z scores for the percent infection are plotted for all 1280 compounds with the replicates on each axis for human cells (Figure 2B) and *Drosophila* cells (Figure 2C). Drugs with a Z score of <−1.7 in duplicate represent our positive candidates for this screen (p<0.002) and are in the lower left quadrants of Figure 2B–C.

### Analysis of nuclei presents a simple counter-screen for cytotoxicity

An important facet of drug screening is the development of counter-screens that allow the identification and removal of artifactual sources of activity, such as compounds that suppress virus infection via cytotoxic effects or by general inhibition of cellular metabolism. Using the information on cell number obtained in this high-content format, we applied the same robust statistics to cell counts, making it possible to identify cytotoxic small molecules in each well (Figure S1). Using this simple strategy, we stratified the candidates into toxic versus non-toxic inhibitors. Drugs were considered toxic if the Z score was less than −2 in duplicate at 10 µM (>20% decrease in cell number). Of the 46 drugs that inhibited infection in 293T cells, 24 showed toxicity while 19 of the 28 drugs identified in *Drosophila* cells were cytotoxic at 10 µM (Table S1). While a significant number of our candidates were toxic at 10 µM, we were able to uncouple viral inhibition from cytotoxicity for a number of inhibitors simply by decreasing the dosage, suggesting that the toxicity was unrelated to the known target specificity (see below and Table 1).

**Table 1:** Inhibitors of RVFV infection.

Drug Name	Drug Class	Cell type in screen	IC50 (µM) 293T	Toxicity uncoupled 293?	IC50 (µM) S2	Toxicity uncoupled S2?
EIPA	Ion Pump	Both	20.6	yes	17.6	not toxic
Chelerythrin Cl	PKC	Both	**	no	6.6	yes
Rottlerin	PKC	Both	2	yes	4.3	yes
Thioridazine	Ca^2+^ Channel	Both	26.2	yes	24.4	not toxic
Thapsigargin	Intracellular Calcium	Both	0.6	yes	0.2	yes
Mibefradil	Ca^2+^ Channel	293T	12.4	yes	8.1	not toxic
Brefeldin A	ER/Golgi transport	293T	0.7	yes	1	not toxic
DEQ	K^+^ Channel	S2	44.3	not toxic	1.4	not toxic
PMA	PKC	293T	NO	not toxic	NO	not toxic
Methotrexate	DNA Metabolism	293T	**	no	94.4	yes
Sanguinarine Cl	Ion pump	293T	4.8	yes	2.9	yes
Tyrphostin A9	CRAC chanel	293T	2.5	yes	1.1	yes
Spiperone	Ca^2+^ Channel	293T	45.9	not toxic	49.7	not toxic
Wortmanin	PI3K	S2	0.2	yes	0.6	yes
U73122	Lipid	S2	**	no	1.4	yes
DMA	Ion Pump	293T	52.2	not toxic	15	not toxic
Vinblastine Sulfate	Microtubules	Both	24.7	yes	0.3	yes
**Drugs used that were not in the screen**						
Ribavirin	Nucleoside analog		41.9		10	
Chloroquine	Acidification		41.2		31.9	
Ammonium Cl	Acidification		310.3		98.5	
CytocholasinD	Actin		**		0.2	
Nocodozole	Microtubules		35.9		47.7	
**PKC inhibitors:**						
GF109203X	pan-PKC		**		NO	
Calphostin C	pan-PKC		1.4		8.9	
Gö6976	Classical PKC		NO		49.3	
PKC Inhib 20–28	Classical PKC		NO		NO	
Ro-332-0432	Classical PKC		NO		NO	

### Candidate inhibitors of RVFV infection

Using this approach we identified 46 drugs that attenuated infection in 293T cells while 28 drugs did so in S2 cells. The larger number of drugs identified in human cells likely reflects the fact that these small molecules were developed for use in mammalian systems. Analysis of the categories of all of the drugs identified revealed 23 classes, as determined by the LOPAC library (Table S1, Figure 2D). Drugs inhibiting ion pumps and the cytoskeleton were significantly overrepresented in the results compared to the representation in the library (p<0.02).

Strikingly, there was a large overlap in the drugs identified that attenuate infection in both cell types, indicating pathways utilized by the virus in both mammals and insects (p<10^−16^; Figure 2E). Analysis of the inhibitors that attenuated infection in both cell types revealed that the inhibitors could be divided into 9 specific categories. Drugs inhibiting PKC, ion pumps, the cytoskeleton and apoptosis inducers were all significantly overrepresented in the data set compared to the library (P<0.02; Figure 2F).

Furthermore, a subset of these drugs are known inhibitors of macropinocytosis, a well-established entry pathway for a variety of viruses [16], [17], [18], [19]. Macropinocytosis is dependent upon PI3K activity, sodium/hydrogen antiporters, as well as an intact cytoskeletal network. It is also dependent upon PKC activity, though the specific PKC isoform(s) important for this process are not known. We identified inhibitors against each of these targets including Wortmannin, a well-known PI3K inhibitor, Rottlerin and Chelerythrine Chloride which are broadly-active PKC inhibitors, and antiporter inhibitors including amiloride derivatives such as EIPA [25]. Although some of the drugs that target PKC or macropinocytosis inhibited infection in only one cell type, we identified some inhibitors that reduced infection in both cell types (Table S1). We were particularly interested in validating those compounds that reduced RVFV MP12 infection in both hosts as they likely target fundamental aspects of RVFV replication.

### Validation of RVFV inhibitors

**Figure 3:**
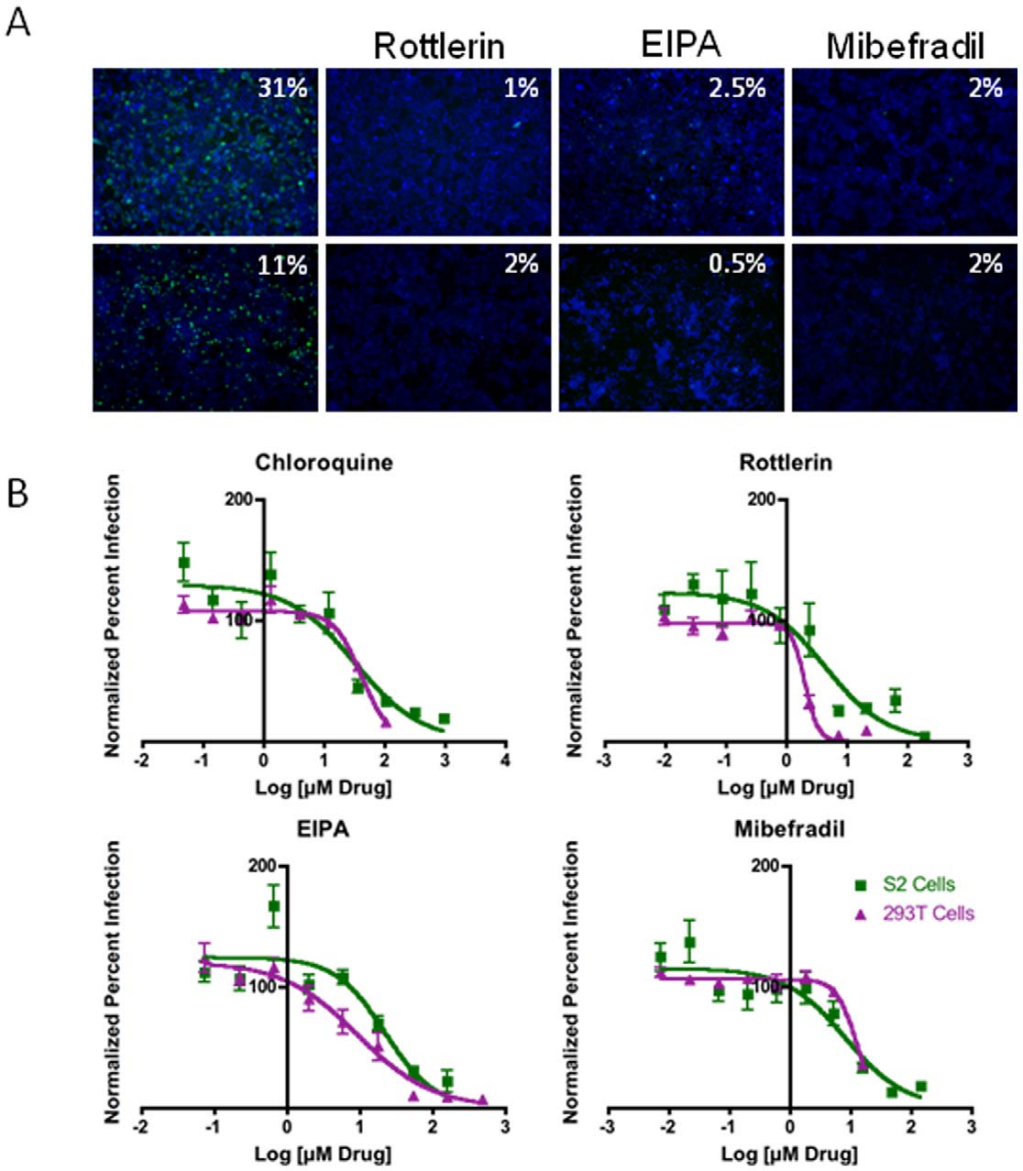
Validation of RVFV candidate inhibitors. **A**. 293T cells were pre-treated with 7 mM Rottlerin, 160 mM EIPA, 16 mM Mibefradil, or vehicle, and infected with RVFV MP12 (MOI = 0.3) for 16 hours and monitored by immunofluorescence with anti-RVFV N (green) and the nuclear dye DAPI (blue). Infected cells were visualized by immunofluorescence and the percent infection quantified as above. **B**. *Drosophila* S2 cells were pre-treated with 21 mM Rottlerin, 53 mM EIPA, 48 mM Mibefradil, or vehicle, and infected with RVFV MP12 (MOI = 0.05) for 48 hours and monitored by immunofluorescence with anti-RVFV N (green) and the nuclear dye DAPI (blue). Infected cells were visualized by immunofluorescence and the percent infection quantified as above. **C**. IC_50_ concentrations for the indicated drugs were determined for mammalian 293T cells and *Drosophila* S2 cells.

To validate our candidates, we re-tested a subset in both mammalian and insect cells (Figure 3, Table 1). We concentrated on small molecule inhibitors that inhibited infection in both cell types as well as the over-represented categories of ion pumps (EIPA), PI3K (Wortmannin), PKC inhibitors (Rottlerin, Chelerythrin Chloride), and calcium inhibitors (Thioridazine, Thapsigargin), many of which are known in mammalian cells to inhibit macropinocytosis. Cytoskeletal inhibitors were also over-represented in our set and included 3 of the 5 microtubule inhibitors in the set and Brefeldin A, an inhibitor of secretion. Inhibitors of the actin cytoskeleton were absent from the library. Altogether, we re-tested 17 candidates listed in Table 1 and found that all but one attenuated infection in one or both cell types, making our false positive rate quite low. Furthermore, we found that all inhibitors efficacious in 293T cells inhibited RVFV MP12 infection in the HeLa and Vero cells (data not shown). We also tested the actin inhibitor Cytochalasin D and control drugs including Ribavirin, Chloroquine and Ammonium Chloride (Table 1), and these too inhibited infection.

IC_50_ values were calculated for inhibitors both in insect and human cells (examples in Figure 3, Table 1). In doing this, we could uncouple cytotoxicity and viral inhibition for a number of drugs, using robust statistical calculations on nuclei as described for the screen to determine cytotoxicity. All six drugs that were cytotoxic in the primary S2 screen remained inhibitory to infection at concentrations that were no longer toxic (Table 1). Nine of the 11 drugs that were cytotoxic in the primary 293T screen were no longer toxic at concentrations that remained inhibitory to infection (Table 1). This is due to the fact that in many cases, toxicity is due to off-target effects that may be affecting another essential protein at a higher concentration than the previously described target. We calculated IC_50_ values only for non-toxic compounds (Table 1). However, some of the drugs remained highly cytotoxic in mammalian cells, significantly reducing the number of cells at all concentrations that were inhibitory to RVFV MP12 infection; thus, IC_50_ values were not calculated for those drugs (denoted by **, Table 1). Importantly, the drugs that did attenuate both cell types did so with similar IC_50_ values suggesting that they are targeting the same cellular factor. These studies showed that our small molecule screen identified a number of anti-RVFV MP12 compounds including multiple inhibitors previously shown to block macropinocytosis.

### Some inhibitors block an early step of RVFV infection

**Figure 4:**
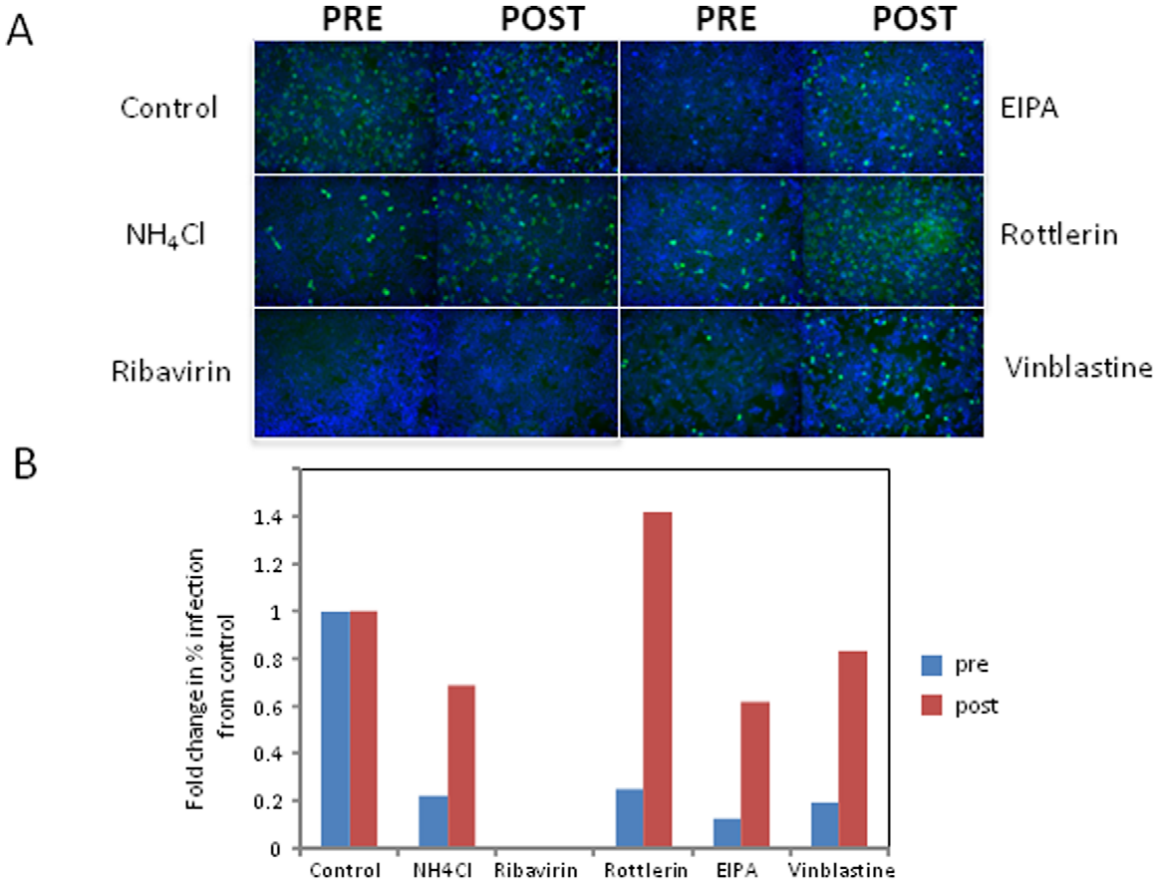
Time-of-Addition Assays. **A**. 293T/17 cells were either pre-treated with the indicated drugs or treated with drugs 1 hour post infection (MOI = 0.3) for 12–15 hours. Representative images from cells treated with NH_4_Cl, Ribavirin, EIPA, Rottlerin and Vinblastine are shown. Cells stained with FITC anti-RVFV N (green) and counterstained with DAPI (blue). **B**. Quantification of the time of addition assay in **A**. was graphed to show the normalized percent infection for the indicated drugs tested. Blue bars are the pre-treatment, and red bars are the one hour post infection treatments.

The lifecycle of RVFV, like that of any virus, involves sequential steps that can be kinetically dissected. Performing time-of-addition experiments can determine if an inhibitor blocks early versus late steps in the lifecycle. We found that binding and entry of RVFV MP12 takes approximately one hour in 293T cells because treatment with lysosomotropic agents such as Ammonium Chloride one hour post-infection no longer blocked infection (Figure 4). In contrast, we found that the nucleoside analog Ribavirin, which inhibits RNA replication, remained inhibitory when added one hour post-infection (Figure 4). Therefore, by treating cells with inhibitors at one hour post-infection, we could determine whether the small molecules targeted an entry or post-entry step in the RVFV MP12 lifecycle.

For these time-of-addition experiments, we tested inhibitors identified in the screen, including two known inhibitors of macropinocytosis, the Na^+^/H^+^ antiporter EIPA and the PKC inhibitor Rottlerin and the microtubule inhibitor Vinblastine. Using this assay we found that all three drugs suppressed RVFV MP12 infection if present at the time of virus addition, but were unable to efficiently inhibit replication when added post-entry (Figure 4). In contrast, the control compound Ribavirin remained inhibitory when added post-entry.

### Protein Kinase C epsilon promotes RVFV infection of mammalian cells

While PKC activity is required for efficient RVFV MP12 infection, it is unclear which gene or isozyme is important for this process. The protein kinase C family comprises 3 classes: cPKC (classical), nPKC (novel), and aPKC (atypical), based on their overall structural similarity and sensitivity to inhibitors [26]. The activity of cPKC isozymes is stimulated by diacylglycerol, calcium, and phosphatidylserine, that of novel isozymes by diacylglycerol and phosphatidylserine, and that of aPKC's by phosphatidylserine. To determine which PKC isozyme class was required for RVFV MP12 infection, we first tested whether RVFV MP12 infection activated calcium signaling. If calcium signaling were activated, it would suggest that a calcium-dependent PKC isozyme might be required. To assay for calcium we used a reporter system that drives the expression of luciferase from the Cre or NFAT promoters in stably or transiently transfected 293T mammalian cells, which are activated by calcium signaling. RVFV MP12 infection did not impact signaling from these promoters (data not shown) [27], [28], making it less likely that the classical PKC isozymes play a central role in the cellular entry of RVFV MP12.

We then tested additional PKC inhibitors that either inhibit all PKC isozymes (Calphostin C) or only the classical PKCs (Gö6976, PKC Inhib 20–28, Ro-332-0432) as characterized in mammalian cells. We found that the pan-PKC inhibitor Calphostin C inhibited infection in both cell types similar to Rottlerin and Chelerythrine Chloride (Table 1). In contrast, Gö6976, PKC Inhib 20–28, or Ro-332-0432 were unable to inhibit RVFV MP12 infection in mammalian cells, while Gö6976 had a weak inhibitory effect in *Drosophila* cells, a cell type that has not been characterized for sensitivity to these inhibitors (Table 1) [29]. These data suggest that the classical PKC isozymes were not essential for RVFV MP12 infection, but that the novel PKC isozymes may promote viral infection.

**Figure 5:**
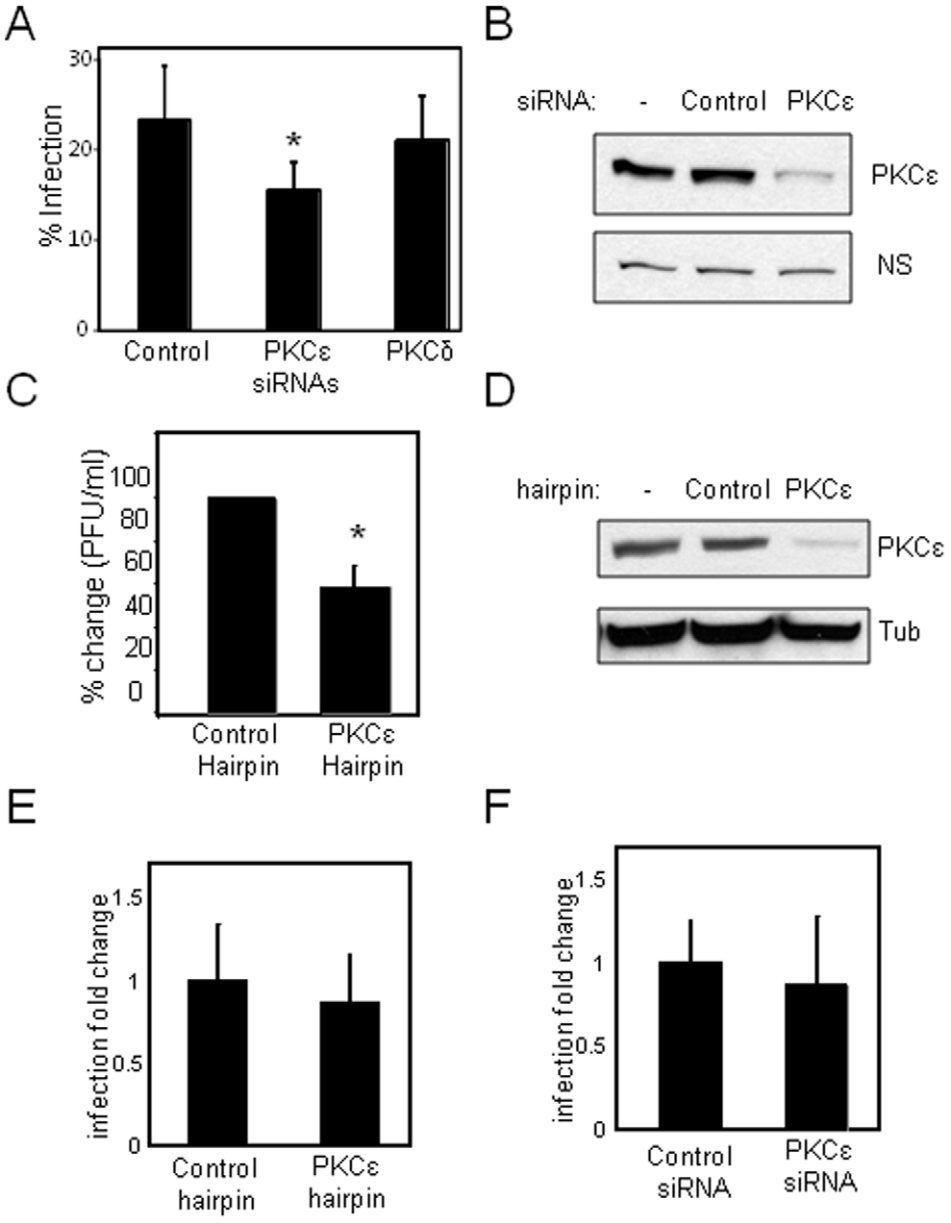
PKCepsilon is required for RVFV infection of human cells. **A**. Human 293T cells were transfected with siRNAs against a control, or the novel PKC isozymes PKCδ or PKCε. The depleted cells were challenged with RVFV MP12 (MOI = 1) for 12 hours were processed for immunofluoresecnce and quantified. Mean±sd for three experiments; * p<0.02. **B**. PKCε is depleted by siRNA treatment as measured by immunoblot compared to a non-specific control (NS). **C**. Human H358 cells stably expressing either a control hairpin or a PKCε specific hairpin were challenged with RVFV MP12 for plaque assays in duplicate for each experiment, averaged, and the fold-change compared to control of the pfu/mL was averaged across three independent experiments; * p<0.02. **D**. PKCε was depleted by RNAi as measured by immunoblot compared to a tubulin control. **E**. Human 293T cells were transfected with siRNAs against a control, or the PKCε and the depleted cells were challenged with poliovirus for 8 hours, processed for immunofluorescence and quantified. Mean±sd for three experiments. **F**. Human H358 cells stably expressing either a control hairpin or a PKCε specific hairpin were challenged with poliovirus for 8 hours, processed for immunofluorescence and quantified. Mean±sd for three experiments.

Because small molecules may inhibit more than one protein, thus having off target effects, we wanted to validate the inhibitor results using an independent approach. To this end, we used RNA interference technology to specifically deplete each of the two major nPKC isozymes, PKCdelta (PKCδ) and PKCepsilon (PKCε). We transiently transfected 293T cells with either a control non-targeting siRNA or a siRNA against PKCδ or PKCε, waited three days for depletion, and challenged the cells with RVFV MP12 for 12 hours. Under these conditions we consistently observed an approximately two-fold decrease in the percentage of infected cells that were depleted for PKCε compared to both the controls and PKCδ-depleted cells at two different MOIs (Figure 5A). Immunoblot analyses demonstrated that the siRNA against PKCε significantly depleted, but did not eliminate, PKCε protein levels compared to untreated or non-targeting controls (Figure 5B).

As an alternative approach to investigate the contribution of PKCε to RVFV MP12 infection, we generated stable hairpin-expressing human adenocarcinoma H358 cell lines that express either a control hairpin or a hairpin against PKCε. We determined the infectivity of these cells by plaque assay and found that there was a significant decrease in plaque number in the PKCε-depleted cells (Figure 5C). Again, we found that PKCε was depleted in the PKCε hairpin-expressing cells compared to either untreated cells or the control hairpin-expressing H358 cells (Figure 5D). To determine whether PKCε is specifically required for RVFV MP12 infection, or generally required for all viruses, we tested whether poliovirus infection was sensitive to depletion of this factor. Poliovirus, a prototypical picornavirus, is a non-enveloped, positive-stranded RNA virus. Poliovirus infection was not impacted by siRNA against PKCε (Figure 5E) nor by the PKCε hairpin in H358 cells (Figure 5F). These data show that suppression of PKCε levels specifically reduces RVFV MP12 infection of mammalian cells although to a lesser extent than the small molecule inhibitors.

### Protein Kinase C epsilon is required for efficient RVFV infection of an insect

**Figure 6:**
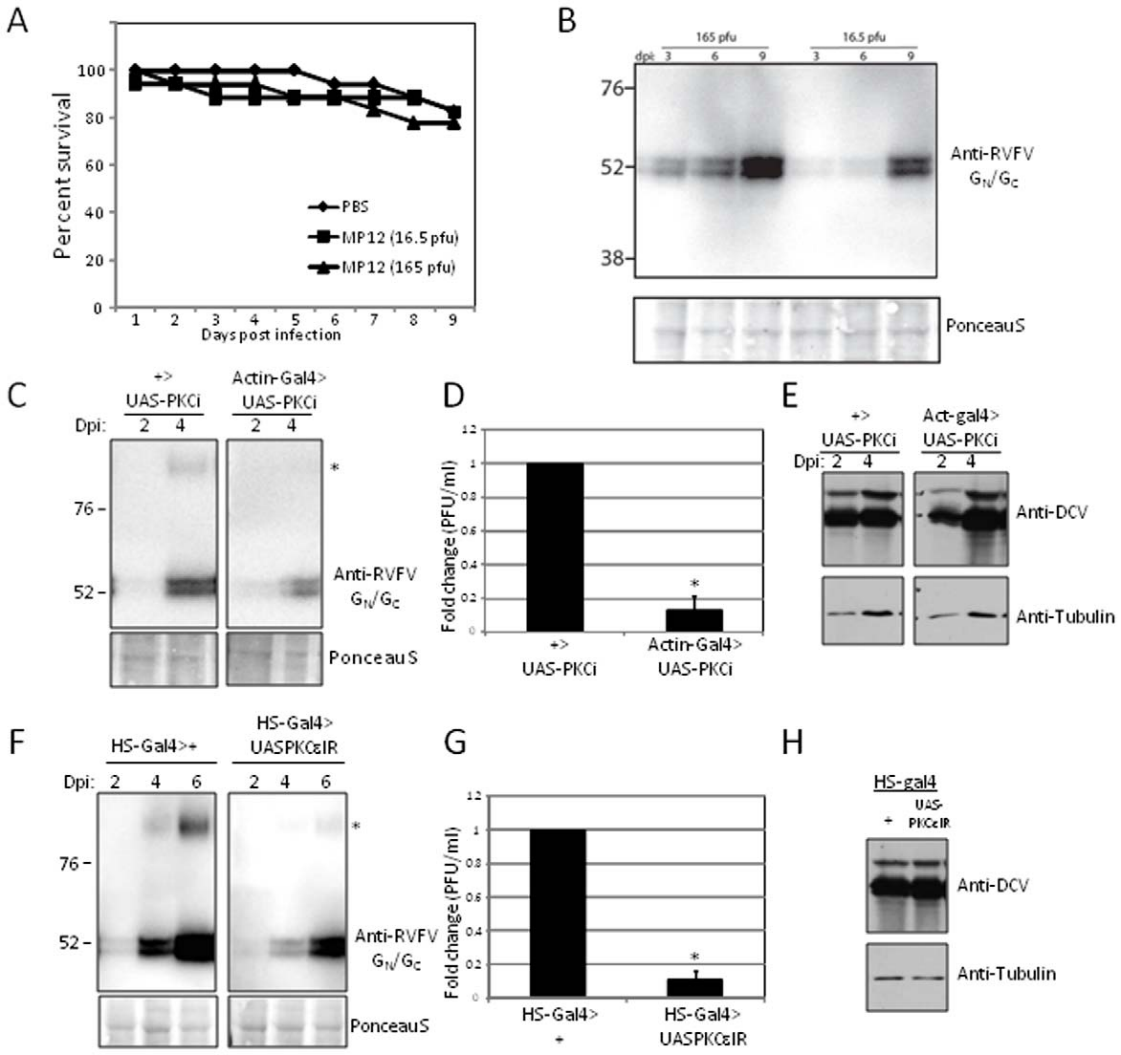
PKCε is required for RVFV infection in adult flies. **A**. RVFV MP12 infection of adult flies is non-lethal. Wild type flies (w1118) were infected with either 165pfu or 16.5pfu or vehicle control (PBS) and monitored daily for lethality. A representative experiment is shown of three independent experiments. **B**. RVFV infection of adult flies can be monitored by an increase in viral antigen production as a function of both dosage and time. Wild type flies were inoculated with the indicated pfu/fly and processed for immunoblot for the glycoproteins G_N_ and G_C_ at the indicated days post infection. A representative experiment is shown of two independent experiments. **C**. Adult flies over-expressing the PKC inhibitor (Actin-Gal4>UAS PKCi) have decreased levels of viral glycoprotein production as measured by immunoblot as compared to sibling controls that do not express the inhibitors (+>UAS PKCi). There are decreased levels of both monomers and dimers (*). A representative experiment is shown of three independent experiments. **D**. Adult flies over-expressing the PKC inhibitor generated and infected as in **C**. have decreased viral progeny production day 4 post infection compared to controls as measured by plaque assay on Vero cells. Replicate experiments are shown. **E**. Adult flies over-expressing the PKC inhibitor (Actin-Gal4>UAS PKCi) have no change in the levels of Drosophila C virus infection as measured by immunoblot against the capsid proteins normalized to control. **F**. Adult flies depleted for the PKC epsilon isozyme (Heat Shock-Gal4>UAS-PKCε IR) have decreased levels of viral glycoprotein production as measured by immunoblot as compared to controls that are not depleted for PKCε (Heat Shock-Gal4>+). There are decreased levels of both monomers and dimers (*). A representative experiment is shown of three independent experiments. **G**. Adult flies depleted for PKCε generated and infected as in **F**. have decreased viral progeny production day 4 post infection compared to controls as measured by plaque assay on Vero cells. Replicate experiments are shown. **H**. Adult flies depleted for the PKC epsilon isozyme (Heat Shock-Gal4>UAS-PKCε IR) have no change in the levels of Drosophila C virus infection as measured by immunoblot compared to controls that are not depleted for PKCε (Heat Shock-Gal4>+).

To study this further at the organismal level, we tested whether RVFV MP12 could successfully infect and replicate in adult flies. As is the case for RVFV infection of their natural mosquito host, infection of *Drosophila* by direct injection of either 16.5 PFU or 165 PFU of RVFV MP12 into the abdominal cavity was non-lethal (Figure 6A). We next tested whether the virus was replicating in the animal. By three days post infection we could detect the production of the viral G_N_ and G_C_ glycoproteins by immunoblot, the levels of which increased as a function of time post infection (Figure 6B). This shows that RVFV MP12 can infect and replicate in flies.

To test the potential role of PKC in infection of an organism, we used the Gal4/UAS system to express a pseudo-substrate inhibitor of PKC (PKCi), which has been previously shown to competitively inhibit different PKC isoforms in adult flies [30]. This peptide inhibits all PKC activity in the fly with an IC_50_ of 0.4 µM. We crossed transgenic flies that can express this specific peptide inhibitor of PKC (PKCi) to transgenic flies that express Gal4 ubiquitously and at high levels downstream of the actin 5C promoter. Over-expression of this transgene does not lead to lethality or a defect in lifespan (data not shown). We challenged flies expressing the PKC inhibitor or sibling controls and monitored the levels of RVFV MP12 replication both by immunoblot and plaque assay analyses post-infection. We found that there was a significant decrease in viral protein production in the PKC inhibited flies (Figure 6C). Furthermore, viral titers were significantly decreased in the PKC inhibited flies (Figure 6D). As a control, we infected flies with Drosophila C virus, a picorna-like virus and a natural pathogen of *Drosophila* [15]. We monitored the levels of Drosophila C virus antigen production by immunoblot as a function of time post-infection and found that there was no change in viral replication in the PKC inhibited flies (Figure 6E). Altogether, these findings are consistent with the PKC inhibitor data that Chelerythrine Chloride, Calphostin C, and Rottlerin inhibit RVFV MP12 infection of *Drosophila* cells (Table 1).

*Drosophila* has homologs for many of the mammalian PKC isozymes including epsilon. Therefore, we next tested whether the *Drosophila* PKCε homolog, PKC98e, was also required in *Drosophila* for RVFV MP12 replication as we demonstrated in human cells. Unlike many of the other PKC isozymes in flies, but similar to mammals, PKCε is expressed ubiquitously and at high levels in the adult (http://flyatlas.org/atlas.cgi?name=FBgn0003093) [31]. We tested a requirement for PKCε through the use of a transgenic strain of flies expressing an inverted repeat or snap-back construct against PKCε. Upon expression of this transgene, a double-stranded RNA is produced which is complementary to PKCε mRNA, and results in RNA interference and depletion of PKCε. Using the Gal4/UAS two-component system we induced expression in adult flies using transgenic animals that express Gal4 under a heat shock promoter. We verified that PKCε was indeed depleted by RT-PCR (Figure S2). Next, PKCε-depleted flies or controls were challenged with RVFV MP12 and monitored again for viral replication. We found that PKCε-depleted flies supported a decreased level of RVFV MP12 replication as measured by viral antigen production (Figure 6F). In addition, viral titers were significantly decreased in PKCε-depleted flies (Figure 6G). In contrast, we found that PKCε-depleted flies supported a similar level of Drosophila C virus replication as measured by viral antigen production (Figure 6H). These data demonstrate that PKCε is specifically required for RVFV MP12 infection in adult flies.

## Discussion

Arboviruses constitute a group of relatively understudied viruses for which there are no therapeutics or vaccines. RVFV, a member of the *Bunyaviridae* family, not only causes significant morbitity and mortality in humans and cattle, but is also considered a Category A bioterrorism agent due to presence of permissive vectors in the United States. Little is known about the cellular pathways and factors co-opted for the replication cycle of RVFV in either the insect or mammalian host. However, the fact that RVFV replicates efficiently in such diverse hosts suggests that it may utilize conserved host factors and pathways as part of its replication cycle. To this end, we first developed a high-throughput assay to monitor infection of the attenuated strain MP12 in both insect cells (*Drosophila* S2 cells) and human cells (293T cells) and used this assay to probe a library of small molecules with known or predicted targets. Using this strategy we set out to uncover essential pathways and gene products subverted by RVFV MP12 for infection. Moreover, by performing this screen in parallel with host cells derived from insects and humans we could readily assess the similarities and differences between the host factor dependencies.

Perhaps surprisingly, we identified a large number of pharmacologically active small molecules that suppressed RVFV MP12 infection in insect and mammalian cells, with many of these targeting molecules involved in signaling pathways. A larger number of compounds suppressed RVFV MP12 exclusively in mammalian cells, but whether this indicates significant differences in how RVFV interacts with mammalian and insect cells or whether the compounds (identified via their abilities to inhibit mammalian targets) fail to interact with their *Drosophila* homologs is not yet known.

Among the inhibitors of RVFV MP12 infection identified in both hosts were several that target PKC. PKC is a family of closely related serine/threonine kinases that regulate diverse processes, with several having been implicated as playing roles in virus infection, dissemination or pathogenesis [32], [33], [34], [35]. There are three classes of PKC isozymes that have distinct cofactor requirements. Therefore, we set out to identify the particular PKC family member was involved in RVFV MP12 infection. Using more specific pharmacological inhibitors we found that the classical PKC isozymes were dispensable, but a novel PKC isozyme was important for infection. The novel PKC isozymes are calcium insensitive, but phospholipid and diacylglycerol-dependent. Although the activation mechanisms between these classes are distinct, the specific function of each isozyme has been difficult to establish because in many cases these kinases have overlapping substrate specificities. Moreover, studies from dominant-negative or constitutively active isozymes have been misleading, necessitating loss-of-function studies. We used RNA interference both in human cell lines and adult fruit flies to demonstrate a specific role for PKCε in promoting RVFV MP12 infection. While the PKC inhibitors routinely inhibited infection by at least 10-fold, RNAi modestly but significantly attenuated infection (∼2-fold). There are a number of possible explanations. First, our knock down was only partial, while the small molecules are more potent. A stronger depletion might reveal a stronger dependence on PKCε. Alternatively, multiple isozymes may contribute to the phenotype, making a single knock-down weaker. Lastly, it is possible that the drugs may be targeting multiple genes that are playing roles in viral infection. While possible, we feel it is unlikely since a number of structurally unrelated inhibitors that target PKC are inhibitory to RVFV replication. The likelihood that the alternate targets of each of these inhibitors are the same is low.

Time-of-addition experiments indicated that PKC activity promoted infection at the time of viral entry, suggesting that intracellular signaling through PKC was facilitating this process. PKC inhibitors have also been shown to block infection with West Nile virus, an arbovirus in the *Flaviviridae* family [35]. In the case of RVFV entry, infection is dependent on its delivery to an acidic cellular compartment as low pH provides the trigger for the conformational changes needed for membrane fusion. However, the precise route taken by RVFV to reach an acidic compartment is not known. Most small, enveloped viruses have been shown to enter cells by either clathrin-mediated or caveolin-mediated endocytosis [16]. Although there has been little work on bunyavirus entry pathways to date, recent studies have revealed that a number of different hantaviruses, a class of bunyaviruses, are dependent upon actin and microtubule networks for entry, as we have shown for RVFV MP12 [36]. PKCε contains an actin binding motif that is unique to this individual member of the PKC family [37] suggesting that this isozyme may be playing a role in this context.

Furthermore, we identified a number of the classic inhibitors used to block macropinocytosis as required for efficient RVFV MP12 infection. Early ultrastructural studies of RVFV infection of Vero cells revealed that virus is taken up in large protrusions, consistent with macropinosomes [38]. Adenovirus, a small DNA virus, utilizes both macropinocytosis and clathrin-mediated endocytosis for infection, triggered by receptor engagement [39]. The virus itself is internalized in clathrin-coated pits, but is unable to enter cells without concomitant activation of macropinocytosis, which facilitates the pH-activated escape from endosomes. In contrast, vaccinia, a large DNA virus, both triggers macropinocytosis and is dependent upon this pathway for entry [19]. Further studies are necessary to determine if there is a role for macropinocytosis in RVFV entry and infection.

We found that when macropinocytosis inhibitors were used or when PKCε expression was suppressed, RVFV MP12 infection still occurred, albeit at significantly reduced levels. Whether residual infection levels under these conditions reflect incomplete blockade of the gene targets or the presence of alternative entry pathways is not clear. However, the fact that RVFV MP12 infection across disparate hosts, including insects and mammals, was significantly reduced by both genetic and pharmacological inhibitors of macropinocytosis and PKC suggests that this is a general requirement for efficient RVFV infection. Furthermore, it implicates PKCε as playing a specific and important role in this process. Importantly, PKCε is dispensable in mice [40], suggesting that this cellular factor may be a suitable target for non-toxic anti-RVFV therapeutics.

By screening a library of known biologically active drugs, this study provides insight into a number of aspects of RVFV infection. First, this strategy allowed us to identify previously unknown inhibitors of the MP12 strain of RVFV. While this is an attenuated strain, it is likely that many of the inhibitors we found will also block infection of wild type RVFV [41], [42]. This alone is an essential starting point to develop more potent therapeutics and creates new biological probes. Second, we found that many of the same cellular pathways that are required for infection in the mammalian host are also required for infection in an insect host. Lastly, by extending our approach to additional medically relevant arthropod-borne viruses for which there are no current therapeutics, we may be able to identify additional inhibitors and targets as a first step toward the control of these pathogens.

## Materials and Methods

### Cells, viruses, antibodies, and reagents

*Drosophila* S2 cells were grown and maintained in Schneiders *Drosophila* media supplemented with 10% FBS (JRH), 100 µg/mL penicillin/streptomycin and 2 mM L-glutamine. Mammalian cells were maintained in DMEM supplemented with 10% FBS (GIBCO) 100 µg/mL penicillin/streptomycin and 2 mM L-glutamine. Vero-E6, 293T/17 and H358 cells are available from ATCC. RVFV strain MP12 was grown in Vero-E6 cells supplemented with 10% FBS (GIBCO). MP12 used to infect *Drosophila* cells was concentrated through a Centricon-70^+^ 30,000 kDa filter. Poliovirus strain Sabin 2 was a kind gift from C. Coyne (U. Pitt) grown and purified as described [43]. Drosophila C virus was grown and purified as described [44]. Mouse monoclonal antibodies to RVFV N (1D8-1-2), G_N_ (7B6-2-2-2) and G_C_ (R2-1F7-3-2) were a gift from C. Schmaljohn (USAMRIID), and rabbit polyclonal antibody to RVFV G_N_ (154) (PROSCI) were used to detect RVFV antigens. Anti-TGN46 (Abcam) and Anti-GM130 (Abcam) were used to detect the Golgi apparatus in mammalian and *Drosophila* cells respectively. Mouse anti-enterovirus VP1 (Ncl-Entero) was used to detect poliovirus. Anti-DCV [44] was used to detect Drosophila C virus. Fluorescently labeled secondary antibodies were obtained from Jackson Immunochemicals or Molecular Probes. The PKC inhibitor set was obtained from (Calbiochem), the LOPAC_1280_ library and all other inhibitors were purchased from Sigma.

### Infections and Immunofluorescence

Cells were infected with the indicated MOI of virus in complete media and fixed and processed for immunofluorescence at the indicated time point post infection. For RVFV MP12 infections, cells were fixed in PBS 4% formaldehyde, washed twice in PBS 0.1% TritonX-100 (PBS-T), and blocked in PBS-T/2% BSA. Primary antibodies were diluted in block, added to cells, and incubated overnight at 4°C. Cells were washed in PBS-T, and incubated in secondary antibody for over one hour at room temperature. Alternatively, FITC conjugated anti-RVFV N was incubated overnight at 4°C. For poliovirus infections, cells were fixed with ice-cold methanol for 5 min followed by permeabilization with 0.1% Triton X-100 in PBS. Cells were incubated with anti-VP1 for 1 h at RT. Cells were counterstained with Hoescht 33342 or DAPI (Sigma). Plates were imaged using an automated microscope (ImageXpress Micro), and quantification was performed using MetaXpress image analysis software.

### Small Molecule Screen

Each compound in the library was added at 10µM per well 1 day after seeding cells in 384 well plates. Mammalian cells were seeded at 6,000 cells 24 hours prior to drug treatment, and infected with RVFV MP12 at MOI = 0.08 for 16 hours, for an average infection of 6%. *Drosophila* cells were seeded at 20,000 cells per well 24 hours prior to drug treatment, and infected with RVFV MP12 at MOI = 0.02 for 48 hours, for an average infection of 15%. The 293T/17 cells were fixed and stained with FITC conjugated anti-RVFV N and counter-stained with DAPI. The *Drosophila* cells were fixed and stained with purified anti-RVFV G_N_ followed by anti-mouse texas red secondary. Next, the cells were stained with FITC conjugated anti-RVFV N and counter-stained with DAPI.

### Screen Analysis

Three sites in each well for each wavelength were imaged at 10× for mammalian cells and 20× for *Drosophila* cells using automated microscopy (ImageXpressMicro). Automated image analysis was used to calculate the number of Dapi^+^ cells and the number of infected cells (FITC^+^, Texas Red^+^). These metrics were used to calculate the percent infection for each site, which was averaged for each well. Percent infection was log-transformed, and the median and interquartile range were used to calculate a robust Z score: (log_10_(%infection)-log_10_(median))/(IQR*0.74) [24]. Outliers were identified if the Z score was <−1.7 in both replicates (p<0.002).

### Drug validation and IC_50_s

Mammalian 293T/17 cells were seeded at 25,000 cells per well and *Drosophila* S2 cells were seeded at 100,000 cells per well in 96 well plates. Drugs were added at 10 µM concentrations for initial validation or as a dilution series for IC_50_ assays for 30 minutes before infection. 293T/17 cells were infected for 12–15 hours at an MOI = 0.3 for an average infection of 20%, and S2 cells were infected at an MOI = 0.05 for 36 hours, for an average infection of 10%. Cells were fixed, stained and imaged as described above. IC_50_ values were calculated using percent infection normalized to no drug controls and set at 100%. Sigmoidal curve fit equations in GraphPad Prism are shown. The IC_50_ values are the average of at least two experiments.

### Time-of-addition

Mammalian 293T/17 cells were seeded at 18,000 cells per well in 96 well plates 48 hours prior to infection. Drugs were added at indicated concentrations 30 minutes prior to infection for pre-treatment samples. Virus was added at an MOI = 0.3 for 1 hour for an average infection of 20%, cells were washed to remove unbound virus, and media was replaced containing drugs in both pre-infection treatment and post-infection treatment conditions. Cells were infected for 12–15 hours, then fixed and processed for immunofluoresence as above. Percent infection was calculated; infected cells with no inhibitor were set to 100% and used to normalize the treated samples for three independent experiments.

### Mammalian RNAi

6,000 293T cells were reverse transfected with 20nM siRNA using HiPerfect according to the manufacturers protocol (Qiagen). 48 hours post-transfection, cells were infected with RVFV MP12 (MOI = 1) for 12 hours. 293T cells were infected with poliovirus at an MOI = 1 for 8 hours. H358 cells were infected with RVFV MP12 for plaque assays or poliovirus at an MOI = 10 for 8 hours for immunofluorescence. siRNAs were purchased from Dharmacon (NonTargeting, PKCε, PKCδ). Hairpin constructs were generated against a control (Dharmacon NTS_C) or PKCepsilon (Dharmacon#849) and were transfected into H358 cells. Puromycin selection resulted in stable cell lines that express the hairpins. Plaque assays were performed on BHK or Vero cells and fixed at three days post infection.

### Fly strains and infections

Flies were grown on standard cornmeal dextrose medium supplemented with dry yeast at room temperature. The following fly strains were used in this study: w1118, Actin-Gal4, Heat shock-Gal4, UAS-PKCi (Bloomington), UAS-PKC98E IR (VDRC). Adult flies of the indicated genotypes were injected with ∼50 nL and monitored daily for lethality or were collected for immunoblot analysis or viral titers performed as above [44].

### Immunoblot analysis

Cells or 4–6 pooled flies were lysed in radioimmunoprecipitation (RIPA) buffer supplemented with a protease inhibitor cocktail (Boehringer). Samples were separated by non-reducing SDS-PAGE for anti-RVFV antibodies, and reducing SDS-PAGE for other antibodies. RVFV antigens were detected with a combination of purified anti- RVFV G_N_ and G_C_. PKCε was detected with anti-PKCε (Santa Cruz). HRP-conjugated secondary antibodies (Jackson Biochemical) and FEMTO SuperSignal Chemiluminescence Reagent were used for visualization.

## Supporting Information

Figure S1 **Drug toxicity can be determined by quantitation of cell number.** Robust Z scores for the nuclei counts in duplicate are plotted for each of the four plates of the LOPAC screen. Each plate is shown with a different symbol. Drugs with a Z<−2.0 in duplicate are considered cytotoxic. A. Mammalian 293T cells. B. *Drosophila* S2 cells.
(TIF)

Figure S2 **PKC98E is depleted by RNAi** ***in vivo*** **.** Semi-quantitative RT-PCR was performed on total RNA purified by Trizol (Invitrogen) from either control (+>UAS-PKCε IR) or depleted (Actin-Gal4>UAS-PKCε IR) flies using primers either against PKCε or control (clathrin heavy chain).
(TIF)

Table S1 **Screen Results for inhibitors of RVFV infection.**
(XLSX)
