# Supplementary figures and images for: Rift Valley Fever Virus Infection of Human Cells and Insect Hosts Is Promoted by Protein Kinase C Epsilon
Source: PLoS One. 2010 Nov 24;5(11):e15483. doi: 10.1371/journal.pone.0015483 (PMC2991366; doi:10.1371/journal.pone.0015483)

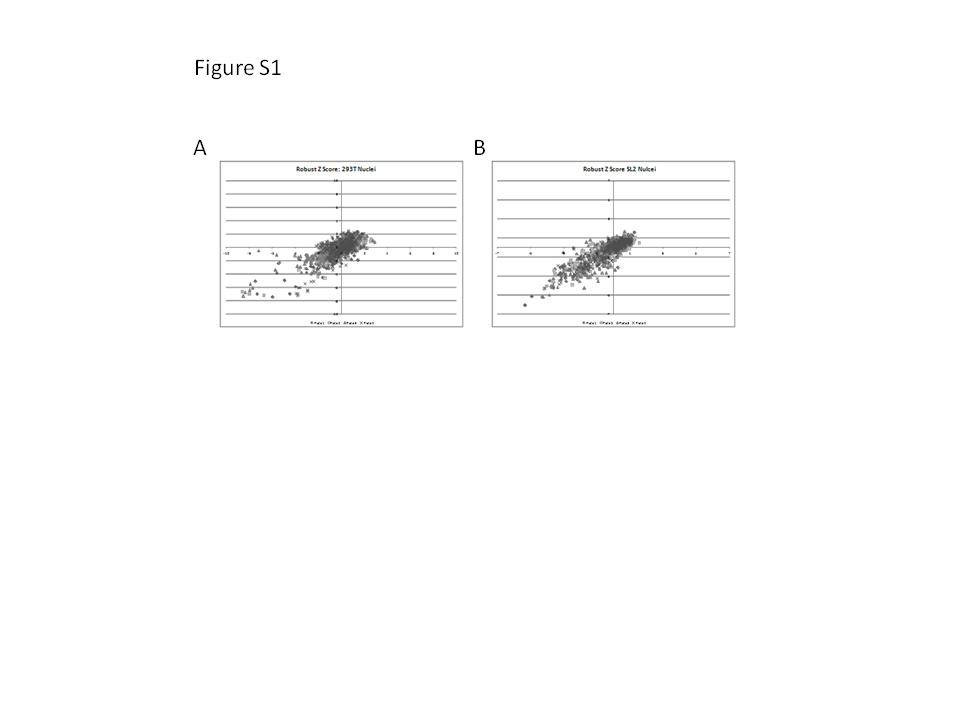

Supplement: Figure S1 — Drug toxicity can be determined by quantitation of cell number. Robust Z scores for the nuclei counts in duplicate are plotted for each of the four plates of the LOPAC screen. Each plate is shown with a different symbol. Drugs with a Z<−2.0 in duplicate are considered cytotoxic. A. Mammalian 293T cells. B. Drosophila S2 cells. (TIF) [file pone.0015483.s001.tif]

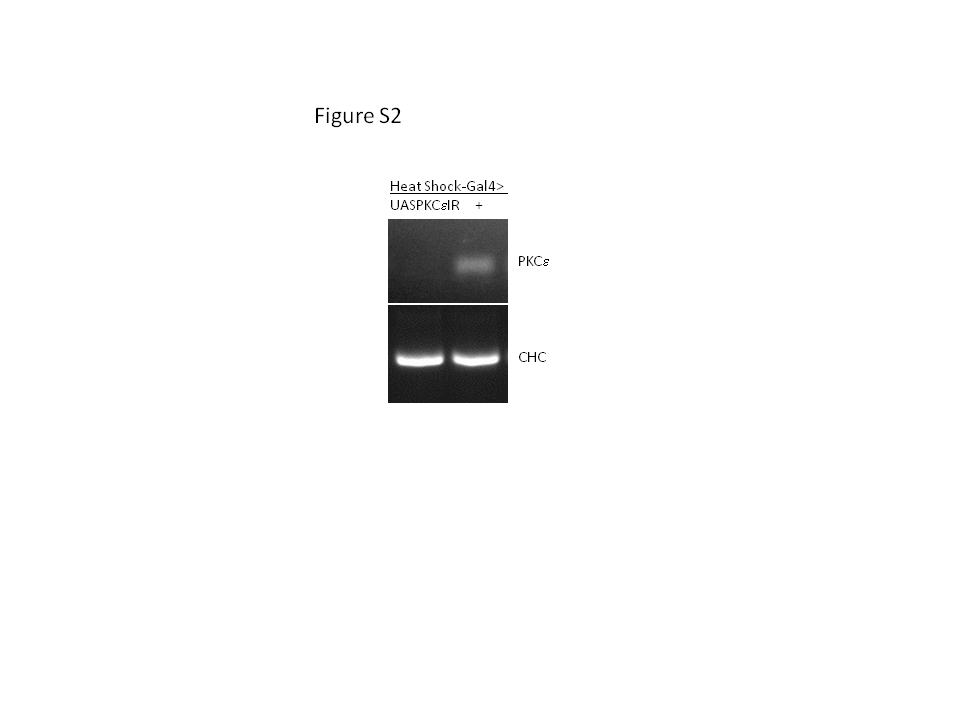

Supplement: Figure S2 — PKC98E is depleted by RNAi in vivo . Semi-quantitative RT-PCR was performed on total RNA purified by Trizol (Invitrogen) from either control (+>UAS-PKCε IR) or depleted (Actin-Gal4>UAS-PKCε IR) flies using primers either against PKCε or control (clathrin heavy chain). (TIF) [file pone.0015483.s002.tif]
